# Fibroblast growth factor 13 deficiency attenuates doxorubicin-induced cardiotoxicity by regulating Parkin-mediated myocardial injury: *Fgf13* deficiency attenuates doxorubicin-induced cardiotoxicity

**DOI:** 10.3724/abbs.2025223

**Published:** 2026-02-09

**Authors:** Jiabing Han, Xuyan Li, Yiming Dong, Yidan Wang, Simeng Lv, Yiyi Zhang, Ran Zhao, Yingke Yan, Yanxue Han, Yu Wang, Jing Yang, Cong Wang, Chuan Wang

**Affiliations:** 1 The Key Laboratory of New Drug Pharmacology and Toxicology Hebei Medical University Shijiazhuang 050017 China; 2 College of Basic Medical Science Hebei University Baoding 071000 China; 3 Zhejiang Key Laboratory of Medical Epigenetics Hangzhou Normal University Hangzhou 310000 China; 4 Department of Pathology and Pathophysiology Hangzhou Normal University Hangzhou 310000 China; 5 Editorial Department of Hebei Medical University Hebei Medical University Shijiazhuang 050017 China; 6 Central Laboratory Affiliated Hospital of Hebei University Clinical Medical College Hebei University Baoding 071000 China; 7 College of Pharmaceutical Sciences Institute of Life Sciences and Green Development Hebei University Baoding 071000 China; 8 Department of Pharmacology Hebei Medical University Shijiazhuang 050017 China

**Keywords:** Keywords: FGF13, cardiotoxicity, Parkin, myocardial injury, apoptosis

## Abstract

The clinical use of doxorubicin (DOX) as a chemotherapeutic agent is limited by its cardiotoxic effects. Fibroblast growth factor (FGF) isoform 13, a distinct type of FGF, has been increasingly recognized as an important regulator of cardiovascular disease. However, its role in doxorubicin-induced cardiotoxicity remains unknown. Therefore, the objective of this study is to investigate the role and mechanism of FGF13 in doxorubicin-induced cardiac injury. C57BL/6 mice are used to establish Dox-induced cardiotoxicity models. The results reveal that mouse weight, cardiomyocyte cross-sectional area, ejection fraction and fractional shortening are decreased in the DOX group. In contrast, *Fgf13* deficiency mitigates doxorubicin-mediated cardiotoxicity, as indicated by increased mouse weight, cardiomyocyte cross-sectional area, ejection fraction and fractional shortening. Mechanistically, the protein expressions of bax and cleaved caspase 3 are elevated in the DOX-treated group, along with decreased JC-1 fluorescence intensity and bcl-2 expression, whereas *Fgf13* knockout prevents these alterations. In addition, Parkin, but not p53, interacts with FGF13 and is upregulated in response to *Fgf13* deficiency in a mouse model of doxorubicin-induced cardiotoxicity. Overall, *Fgf13* knockout attenuates doxorubicin-induced cardiomyocyte apoptosis and mitochondrial damage through the modulation of Parkin, indicating that FGF13 may serve as a promising therapeutic target for DOX-induced cardiotoxicity.

## Introduction

Doxorubicin (DOX), an anthracycline-class drug, is widely used as a chemotherapeutic agent in various malignancies [1]. However, its dose-dependent cardiotoxicity leads to irreversible myocardial degeneration, significantly restricting its clinical application [ 2, 3] . Moreover, DOX-induced cardiotoxicity can manifest both acutely and years after anthracycline treatment, leading to left ventricular dilation and systolic dysfunction, cardiomyocyte apoptosis, and myocardial damage and atrophy [ 2, 4] . Optimizing the options for chemotherapy while avoiding cardiac complications remains a key priority in clinical care. Therefore, further efforts to understand the underlying mechanisms involved in this condition are essential for the development of new therapies.

Fibroblast growth factor (FGF) homologous factors (FHFs) are encoded by four genes within the FGF superfamily (FGF11-14). As part of the FGF superfamily, FHFs do not function as growth factors and are incapable of activating FGF receptors [5]. FGF13, the most abundantly expressed FHF in the murine heart [6], directly binds to microtubules, participates in regulating calcium signaling in heart failure [ 7, 8] , and modulates ROCK signaling in cardiac fibrosis [9]. Moreover, *Fgf13* deficiency enhances caveolae-mediated cardioprotection [10] and inhibits NF-κB activation during cardiac pressure overload [11]. These studies suggest that FGF13 is a potentially significant regulator of heart disease. However, its effect on DOX-induced injury remains unexplored.

Parkin, a RING-between-RING E3 ubiquitin ligase, participates in various complex cellular processes, including mitophagy, apoptosis, immune signaling, and differentiation [ 12, 13] . Moreover, the FGF13-sensitive alteration of Parkin safeguards mitochondrial homeostasis in the endothelium in type 2 diabetic nephropathy through the promotion of mitophagy and the inhibition of apoptosis [14]. Although insights into the molecular mechanism involved in DOX-induced cardiotoxicity are increasing, it remains unclear whether *Fgf13* deficiency mitigates this toxicity through the regulation of Parkin.

Here, we found that cardiac-specific knockout of *Fgf13* ameliorated cardiac dysfunction caused by doxorubicin. Moreover, *Fgf13* deficiency attenuated DOX-induced myocardial atrophy and remodeling. Furthermore, *Fgf13* deficiency alleviated DOX-induced cardiomyocyte apoptosis and mitochondrial damage. Finally, FGF13 modulated Parkin to regulate apoptosis and mitochondrial integrity in the context of DOX-induced myocardial injury, suggesting that FGF13 may be a new therapeutic target for DOX-induced cardiotoxicity.

## Materials and Methods

### Animals

The animals used in this study were adult male C57BL/6J mice aged 8 to 16 weeks. All animal procedures were approved by the Experimental Animal Ethics and Welfare Committee of Hebei Medical University (approval number: IACUC-Hebmu-2022011). The study strictly obeyed the guidelines set by the committee.

The generation of cardiac-specific *Fgf13* conditional knockout mice has been described previously [15]. The mice were allowed to recover for at least one week following tamoxifen induction prior to experimentation.

### Echocardiography

Cardiac function was evaluated via a Vevo® 2100 small animal ultrasound imaging system (FUJIFIUM VisualSonics Inc., Toronto, Canada). Transthoracic two-dimensional (2D)-guided M-mode echocardiography was performed to measure several parameters, including LV ejection fraction (EF%), fractional shortening (FS%), left ventricular end-systolic diameter (LVIDs) and left ventricular end-diastolic diameter (LVIDd).

### Serum biochemical indexes

Serum levels of cardiac injury markers (lactate dehydrogenase, LDH; creatine kinase isoenzymes, CK-MB; creatine kinase, CK) were measured with an automatic biochemical analyzer (SySMES, Kobe, Japan).

### Histological staining

First, cardiac tissues were fixed in 4% PFA at 4°C overnight. The samples were subsequently dehydrated, infiltrated, embedded in paraffin, cut into 3-μm-thick tissue sections via a pathological microtome and mounted on slides. Finally, hematoxylin and eosin (HE) staining was subsequently performed, and the images were analyzed via a light microscope (Olympus, Tokyo, Japan).

### Wheat germ agglutinin (WGA) staining

To analyze the cross-sectional area of cardiomyocytes, heart tissues were stained with WGA (Sigma Aldrich, St Louis, USA) according to the manufacturer’s protocol. Briefly, heart sections were permeabilized with 0.1 % Triton X-100 in PBS for 30 min and washed with PBS. The sections were stained with WGA working solution for 2 h at 37°C and washed with PBS. The nuclei were stained with DAPI (Seven, Beijing, China). Stained sections were scanned via a laser confocal microscope (Leica, Wetzlar, Germany). ImageJ software was used to estimate the cross-sectional area of the cardiomyocytes.

### Quantitative real-time PCR

Total RNA was extracted via the TRIzol reagent (Invitrogen, Waltham, USA). Total RNA (1000 ng) was reverse transcribed into cDNA via the PrimeScriptTM RT reagent Kit with the gDNA Eraser (perfect real-time) Kit (Takara, Osaka, Japan). Quantitative PCR was performed via SYBR (Takara) and gene-specific primers on the CFX96 Touch Real-Time PCR Detection System (Bio-Rad Laboratories, Hercules, USA). The 2 ^–ΔΔCT^ method was used to calculate the relative gene expression, and *GAPDH* was used as an internal control. The primers used were as follows: *Fgf13*, forward: 5′-GAGCCTCAGCTTAAGGGTATAG-3′; reverse: 5′-GTTCAGACCTAGATACCACC-3′; *Cytochrome C*, forward: 5′-GAGGCAAGCATAAGACTGGA-3′; reverse: 5′-TACTCCATCAGGGTATCCTC-3′; *GAPDH*, forward: 5′- *TGTCAGCAATGCATCCTGCA*-3′; reverse: 5′-CCGTTCAGCTCTGGGATGAC-3′.

### TUNEL assay

The tissue sections were dewaxed and rehydrated following standard protocols. The protease K (SolarBio, Beijing, China) solution was prepared at a ratio of 1:100 and diluted with PBS. Each sample was supplemented with 100 μL of the diluted protease K solution and incubated at room temperature for 10–20 min. Then, the blocking solution (5% goat serum in PBS) was added, and the mixture was incubated for 1 h at room temperature. The TUNEL reagent (Proteintech, Wuhan, China) was applied, and the samples were incubated for 1 h at 37°C in the dark. After the samples were washed with PBS three times (5 min each), DAPI dye solution was added, and the samples were incubated at room temperature for 10 min. The fluorescence signals were captured via the laser confocal microscope. The apoptotic index was calculated as the percentage of TUNEL-positive nuclei to DAPI-stained nuclei.

### Measurement of the mitochondrial membrane potential

Mitochondria were extracted from heart tissue via a tissue mitochondrial isolation kit (Beyotime, Shanghai, China). The mitochondrial membrane potential (MMP) was assessed via a JC-1 staining assay kit (Beyotime). In accordance with the manufacturer’s instructions, the purified mitochondria were incubated with JC-1 reagent for 20 min. The mixture was placed in a black 96-well plate and detected with a fluorescence microplate reader (BMG LABTECH, Offenburg, Germany).

### Western blot analysis and coimmunoprecipitation (Co-IP)

Total proteins were isolated from hearts or cells via RIPA lysis buffer (Seven, Beijing, China) supplemented with protease inhibitors (MCE, Monmouth Junction, USA). A BCA protein assay kit (SolarBio, Beijing, China) was used to determine the protein concentration of the lysates. Proteins (50 μg) were separated by 12% or 10% SDS-PAGE, and transferred to PVDF membranes (Millipore, Billerica, USA). After the membranes were blocked with 5% nonfat milk for 2 h, they were incubated with primary antibodies overnight at 4°C. Primary antibodies against GAPDH (1:10,000; Proteintech), FGF13 (1:300; Yenzym, Brisbane, USA), bax (1:1000; Proteintech), bcl-2 (1:1000; Proteintech), cleaved caspase 3 (1:500; Cell Signaling Technology, Boston, USA), p53 (1:1000; Proteintech), Parkin (1:1000; Proteintech) and β-actin (1:5000; Proteintech) were used. The fluorescently labeled secondary antibodies (1:10,000; LI-COR, Lincoln, USA) were subsequently incubated with the membranes at room temperature for 2 h. An Odyssey Infrared Imaging System (LI-COR) was used to analyze the results of the western blot strips.

For coimmunoprecipitation, the protein lysate (500 μL) was incubated with 6–10 μg of antibody or IgG (as a control) at 4°C overnight. The next day, the sample was incubated with 20 μL of protein A/G magnetic beads (Beyotime) for 2 h before being washed again. After that, 100 μL of buffer was added to the sample, which was subsequently heated at 95°C for 5 min. Finally, the samples were subjected to western blot analysis.

### Statistical analysis

All the statistical analyses were performed via GraphPad Prism 8 and SPSS version 20. The data are presented as the mean ± SD. Differences between two groups were analyzed by Student’s *t* test, and differences among multiple groups were analyzed with one-way ANOVA followed by Bonferroni correction for multiple comparisons. *P* values of < 0.05 were considered statistically significant.

## Results

### *Fgf13* deficiency attenuates DOX-induced cardiac dysfunction in mice

**Figure 1:**
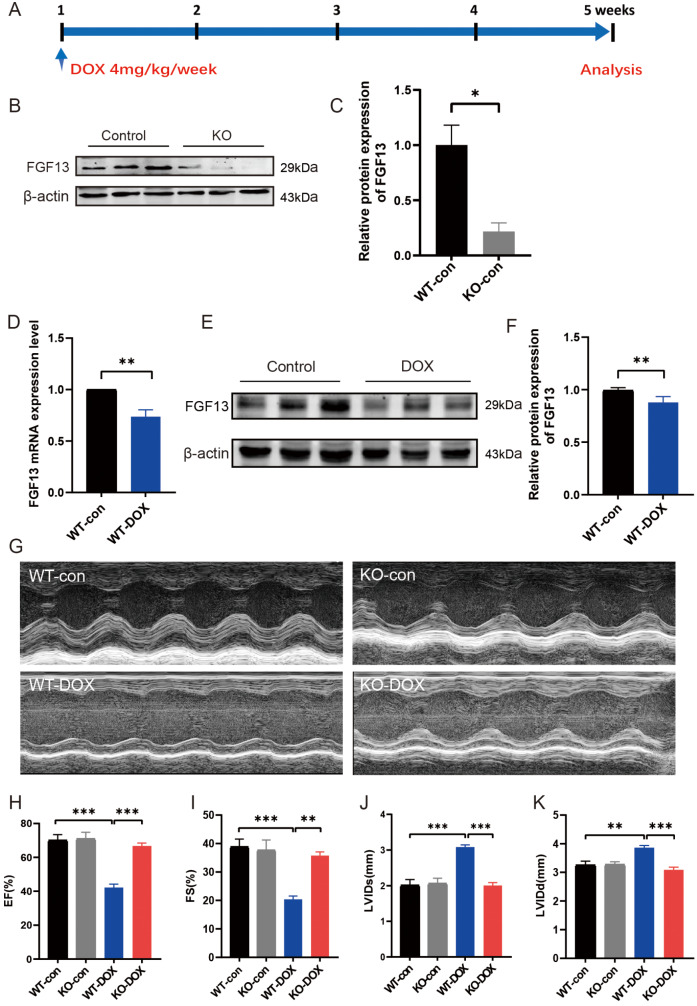
Figure 1 *Fgf13* deficiency attenuates DOX-induced cardiac dysfunction in mice (A) Administration modes in mice. (B) Representative images of FGF13 protein levels in wild-type and Fgf13-knockout mice. (C) Quantitative data of the FGF13 protein (n = 3 in each group). (D) qPCR analysis of Fgf13 mRNA expression in myocardial tissues (n = 6 in each group). (E) Representative images of FGF13 protein levels in myocardial tissues. (F) Quantitative data of FGF13 expression in mice. G) Representative M-type echocardiograms of wild-type control mice (WT-con), cardiac Fgf13-knockout mice (KO-con), wild-type mice treated with DOX (WT-DOX), and Fgf13-knockout mice treated with DOX (KO-DOX). (H-K) Cardiac function was quantified, including ejection fraction (EF), fraction shortening (FS), left ventricular end-systolic diameter (LVIDs) and left ventricular end-diastolic diameter (LVIDd) (n = 5 in each group). *P < 0.05, **P < 0.01, and ***P < 0.001.

To explore whether FGF13 plays a potential role in DOX-induced cardiotoxicity, we generated *Fgf13* conditional knockout (KO) mice via tamoxifen injection. Western blot analysis revealed efficient deletion of FGF13 in cardiac tissue ( Figure 1B,C). We then established a mouse model of chronic DOX-induced cardiotoxicity via intraperitoneal injection of DOX (4 mg/kg once a week for 4 weeks) ( Figure 1A) and detected the expression of FGF13 in mouse hearts. Both western blot ( Figure 1D,E) and qPCR ( Figure 1F) analyses demonstrated that the expression of FGF13 was significantly decreased after DOX injection. To assess the functional consequences, cardiac function was evaluated via echocardiography. Compared with control mice, *Fgf13*-KO mice showed no differences in ejection fraction (EF) ( Figure 1G,H), fractional shortening (FS) ( Figure 1I), left ventricular end systole (LVIDs) ( Figure 1J), or diastolic diameter (LVIDd) ( Figure 1K). However, after DOX injection, *Fgf13* KO increased the FS and EF and decreased the LVIDs and LVIDd compared with those in the DOX group ( Figure 1H–K). Taken together, these findings suggest that *Fgf13* deficiency improves cardiac function in a DOX-induced cardiotoxicity model.

### *Fgf13* knockout inhibits DOX-induced myocardial injury

**Figure 2:**
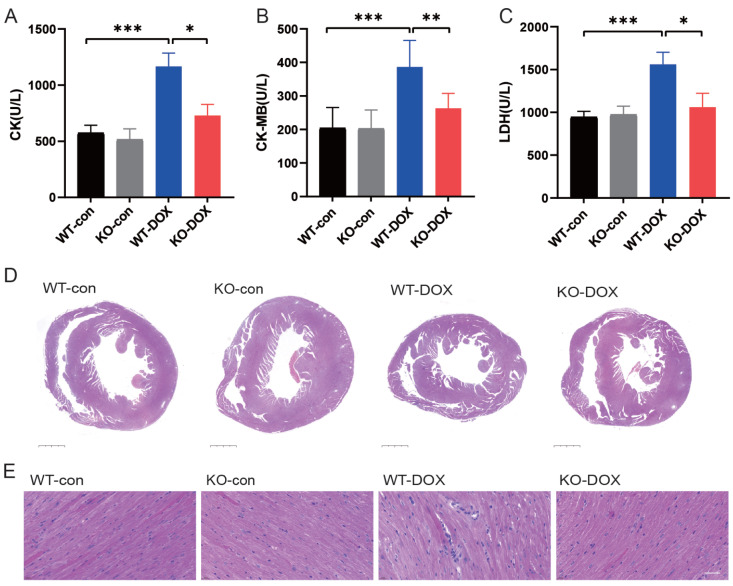
Figure 2 *Fgf13* knockout inhibits DOX-induced myocardial injury (A–C) CK, CK-MB, and LDH activity in mouse serum (n = 9 in each group). (D–E) Schematic diagram of HE staining of the heart cross-sectional area for each group (n = 3 in each group). *P < 0.05, **P < 0.01, and ***P < 0.001.

DOX induces cardiomyocyte atrophy and injury [16], whereas *Fgf13* KO has a cardioprotective effect on several cardiac diseases [ 8, 10, 15] . To evaluate its potential protective effect against Dox-induced myocardial injury, we generated *Fgf13* conditional KO mice and assessed myocardial injury after treatment with DOX. As expected, DOX injection led to significant increases in the serum CK ( Figure 2A), CK-MB ( Figure 2B) and LDH ( Figure 2C) levels. This increased injury response to doxorubicin was significantly attenuated by *Fgf13* KO. Similarly, HE staining revealed that the enlarged intercellular gaps and misaligned muscle fibers in DOX-treated hearts were reversed by cardiac-conditioned *Fgf13* KO ( Figure 2D,E). Overall, these results demonstrated that *Fgf13* knockout in mouse hearts can reduce DOX-induced myocardial injury.

### *Fgf13* knockout inhibits DOX-induced myocardial atrophy

**Figure 3:**
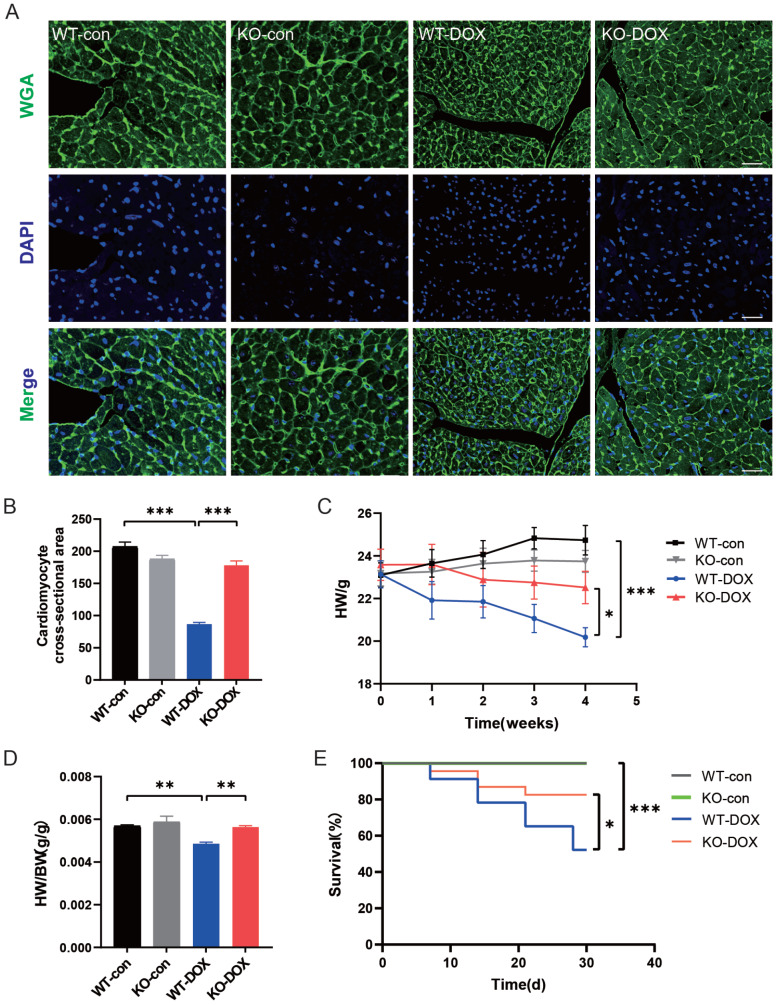
Figure 3 *Fgf13* knockout inhibits DOX-induced myocardial atrophy (A) Schematic diagram of representative heart tissue WGA staining for each group of mice (n = 3 in each group). (B) Quantification of the cardiomyocyte cross-sectional area after WGA staining (n = 3 in each group). (C) Body weight statistics of the mice in each group. (D) Heart weight/body weight ratios (n = 5 in each group). (E) Survival curves after doxorubicin treatment in WT or Fgf13-KO mice (n = 20 in each group). *P < 0.05, **P < 0.01, and ***P < 0.001.

Given that myocardial atrophy is a key component of DOX-induced cardiotoxicity, we next performed WGA staining and measured mouse weight. The results revealed that the mice subjected to DOX injection presented a decrease in the cardiomyocyte cross-sectional area ( Figure 3A,B), body weight ( Figure 3C) and heart weight-to-body weight ratio ( Figure 3D). In contrast, *Fgf13* KO significantly attenuated these atrophic changes, leading to an increased cardiomyocyte cross-sectional area ( Figure 3A,B), preserved body weight ( Figure 3C), and increased heart mass ( Figure 3D) compared with those of the DOX-treated controls. Moreover, owing to the protective effects of *Fgf13* KO on the heart, *Fgf13*-KO mice treated with DOX presented a significantly greater survival rate ( Figure 3E) than the DOX-treated mice ( Figure 3E). Collectively, these data indicate that *Fgf13* knockout in mouse hearts can attenuate DOX-induced myocardial atrophy.

### *Fgf13* deficiency relieves DOX-induced cardiomyocyte apoptosis and mitochondrial damage

**Figure 4:**
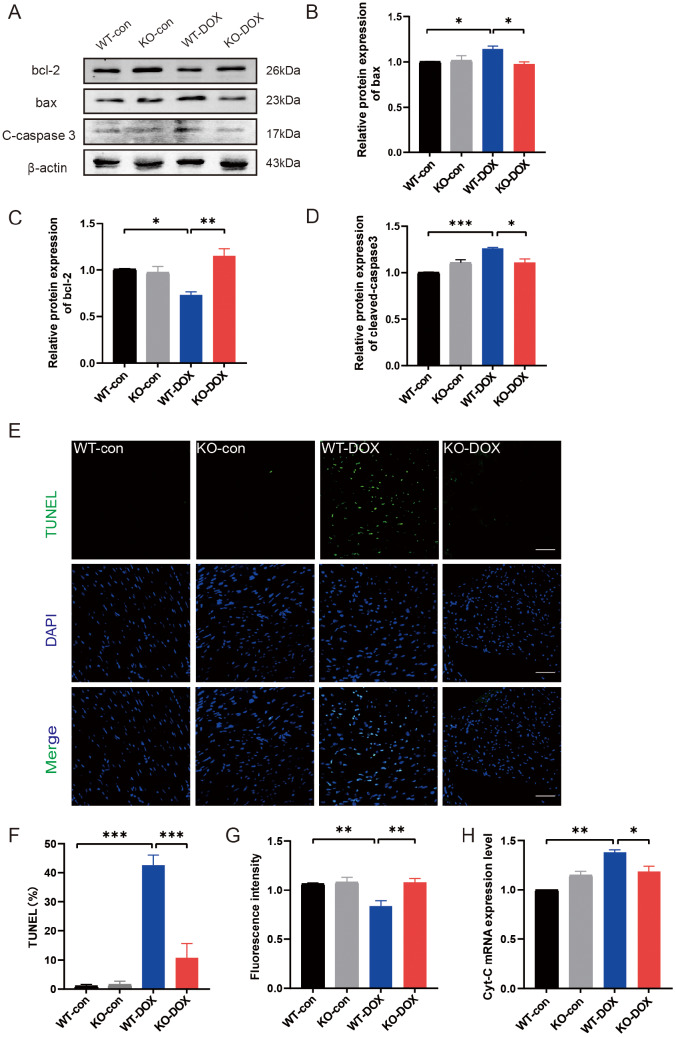
Figure 4 *Fgf13* deficiency inhibits DOX-induced cardiomyocyte apoptosis (A) Representative western blot results showing bax, bcl-2 and cleaved caspase 3 protein levels in the heart. (B–D) Quantification of the levels of apoptotic proteins (n = 3 in each group). (E) Representative TUNEL staining image of a myocardial section. (F) Results from the quantitative analysis of the nuclei of apoptotic cells in mouse hearts, as evaluated by TUNEL staining (n = 3 in each group). (G) Quantitative data of the JC-1 red-to-green fluorescence intensity ratio (n = 3 in each group). (H) Cyt-C mRNA levels in myocardial tissues (n = 3 in each group). *P < 0.05, **P < 0.01, and ***P < 0.001.

Cardiomyocyte apoptosis and mitochondrial damage are pivotal pathological processes of DOX-induced myocardial injury. Therefore, we next explored whether *Fgf13* KO prevents cardiomyocyte apoptosis and mitochondrial damage. The results revealed that the protein expression of the apoptosis regulators bax and cleaved caspase 3 (proapoptotic markers) increased and bcl-2 (antiapoptotic marker) decreased in the DOX group ( Figure 4A–D), and the percentage of terminal deoxynucleotidyl transferase dUTP nick end labeling (TUNEL)-positive cardiomyocytes showed an increasing trend ( Figure 4E,F). Notably, *Fgf13* deficiency ameliorated DOX-induced cardiomyocyte apoptosis by decreasing bax and cleaved caspase 3 expression, increasing bcl-2 expression, and decreasing the number of TUNEL-positive cardiomyocytes. Similarly, compared with the DOX group, the *Fgf13* KO group presented increased mitochondrial membrane potential (detected as JC-1, Figure 4G) and reduced cytochrome C release ( Figure 4H) induced by DOX. These results indicate that *Fgf13* knockout in hearts can ameliorate DOX-induced cardiomyocyte apoptosis and mitochondrial damage.

### *Fgf13* regulates cardiomyocyte apoptosis by modulating Parkin in DOX-induced myocardial damage

**Figure 5:**
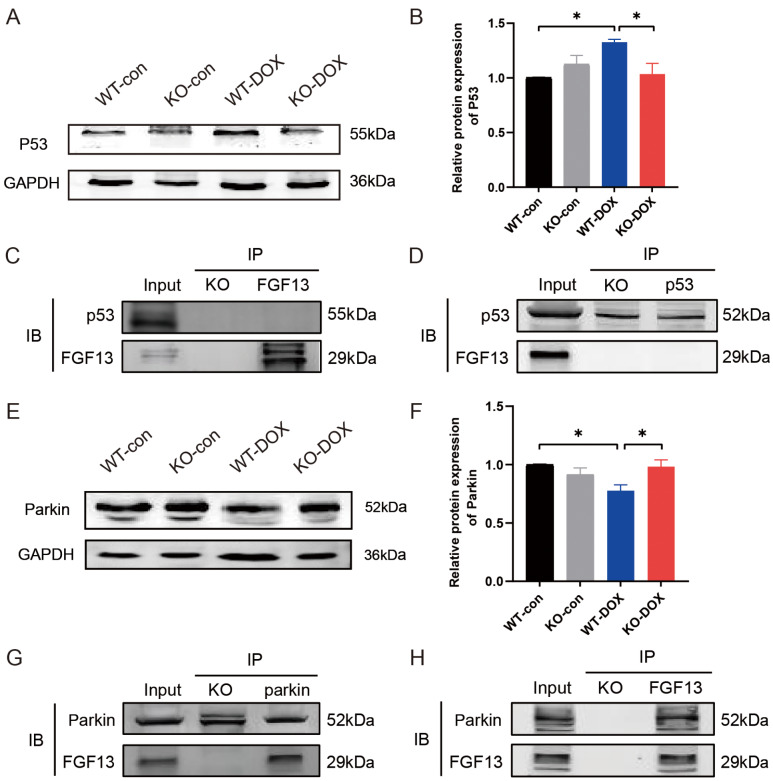
Figure 5 Interactions between FGF13, p53 and Parkin (A) Representative western blot results of p53 protein expression in the heart (n = 3 in each group). (B) Quantitative data of p53 expression in mice (n = 3 in each group). (C,D) Coimmunoprecipitation (Co-IP) assay confirmed the interaction between FGF13 and p53 in myocardial whole-cell lysates. (E) Representative western blot results of Parkin protein expression in the heart. (F) Quantitative data of Parkin expression in mice (n = 3 in each group). (G,H) The ability of FGF13 to bind to Parkin was confirmed by Co-IP. *P < 0.05, **P < 0.01, and ***P < 0.001.

To investigate the mechanism by which FGF13 regulates cardiomyocyte apoptosis, we performed western blot analysis and Co-IP experiments. The results revealed that *Fgf13* KO downregulated p53 ( Figure 5A,B), despite the absence of a direct physical interaction between FGF13 and p53 ( Figure 5C,D). We subsequently examined the interaction between FGF13 and Parkin in DOX-induced myocardial damage. Co-IP assays revealed the interaction between FGF13 and Parkin in whole-cell cardiac lysates ( Figure 5G,H). Moreover, compared with WT mice treated with DOX, mice with *Fgf13* deficiency showed upregulated Parkin expression following DOX treatment ( Figure 5E,F). Overall, these results suggest that the protective effect of *Fgf13* deficiency against cardiomyocyte apoptosis is associated with the modulation of Parkin.

## Discussion

DOX remains an effective and widely used broad-spectrum chemotherapeutic agent. However, its clinical application is limited by severe dose-dependent cardiotoxicity [4]. The FGF family has gained increased interest owing to its cardioprotective effects [ 6, 8, 10] . However, FGF13, an intracellular protein lacking a signal peptide for secretion and function, has remained unexplored in the context of DOX-induced cardiotoxicity. Therefore, the pertinent new finding of this study is that conditional *Fgf13* KO protects mice against DOX-induced cardiotoxicity, weight loss, and increased mortality. These protective effects are achieved by mitigating DOX-induced cardiomyocyte apoptosis and mitochondrial damage. Importantly, the protective effect of *Fgf13* deficiency on cardiomyocyte apoptosis is associated with Parkin. In addition, Parkin, but not p53, interacted with FGF13 and was upregulated by *Fgf13* deficiency in a mouse model of DOX-induced cardiotoxicity. This study revealed that *Fgf13* KO attenuates cardiomyocyte apoptosis and mitochondrial damage through the modulation of Parkin, thereby alleviating DOX-induced cardiotoxicity.

DOX administration can lead to myocardial injury and dysfunction, which are typically assessed via echocardiography [ 17, 18] . Moreover, circulating biomarkers offer sensitive, personalized, and cost-effective surveillance in patients receiving anthracycline therapy [19]. Consistently, our study revealed that DOX-treated mice exhibited a progressive decline in diastolic function; increased serum biomarkers (such as CK, CK-MB and LDH); and a decreased survival rate compared with those in the control group. FGF13, the most abundant FHF in the heart, has been reported to exert protective effects under conditions of cardiac pressure overload [11]. However, its role in DOX-induced cardiotoxicity has not been previously elucidated. Our study revealed that *Fgf13*-KO mice presented increased contractile function and survival rates and decreased myocardial injury in response to DOX, indicating that *Fgf13* conditional KO in the heart resulted in significant cardioprotection after DOX injection.

Apoptosis represents the most researched programmed cell death pathway in anthracycline-induced cardiotoxicity [20]. DOX administration increases oxidative stress, which activates heat shock protein and stabilizes the p53 protein, which is responsible for the generation of proapoptotic factors [21], and eventually causes increased posttranslational modifications of Bcl-2 [22] and the induction of caspase-3 [23], which causes apoptosis. Although we have already established that *Fgf13* deficiency confers protection against doxorubicin-induced myocardial injury, the involvement of apoptosis in this protective mechanism remains elusive. To address this question, we conducted further experiments. Our study revealed that *Fgf13* deficiency ameliorated DOX-induced cardiomyocyte apoptosis, as evidenced by increased Bcl-2 expression, decreased Bax and cleaved caspase-3 expression, and reduced numbers of TUNEL-positive cardiomyocytes. Collectively, these findings suggest that *Fgf13* deficiency alleviates DOX-induced cardiomyocyte apoptosis.

Mitochondrial damage plays a significant role in cardiomyocyte apoptosis [24]. Several studies have shown that DOX can accumulate in the mitochondria [25], disrupting the integrity of the mitochondrial membrane and triggering the release of cytochrome C [26], which subsequently activates the intrinsic apoptotic pathway and cell death [27]. Consistent with these findings, our experiments revealed that, compared with control mice, DOX-treated mice presented decreased JC-1 fluorescence intensity and increased cytochrome C expression. However, *Fgf13* KO increased the mitochondrial membrane potential and reduced DOX-induced cytochrome C release, suggesting that *Fgf13* KO inhibits apoptosis by improving mitochondrial dysfunction.

As a key inducer of cell death [28], p53 promotes the expression of a number of genes involved in apoptosis, including proapoptotic members of the Bcl-2 family [29], and typically triggers apoptosis via mitochondrial release of cytochrome C and caspase activation [30]. To verify the mechanism by which *Fgf13* KO inhibits apoptosis, we examined the relationships between p53 and FGF13. Despite the lack of a direct interaction, as determined by Co-IP, p53 expression was downregulated in DOX-treated *Fgf13*-KO mice. Interestingly, previous studies have reported that miR-504, along with its host gene *Fgf13*, is subject to constitutive transcriptional repression by p53. However, these studies highlight the impact of FGF13 1A on the transcription and processing of rRNA in cancer [ 31, 32] . These observations indicate that p53 downregulation signaling could be intricately regulated.

A previous study revealed another function of Parkin as a transcriptional repressor of p53 [33]. Therefore, we continued to examine the relationship between FGF13 and Parkin. Parkin, a critical mediator of mitochondrial quality control, has been implicated in determining cell fate upon mitochondrial damage [ 34– 36] . Moreover, Parkin has been identified as an important target of FGF13 in regulating mitochondrial damage caused by diabetic nephropathy [14]. Consistent with previous reports, in our study, Parkin interacted with FGF13 in the heart and was upregulated by *Fgf13* deficiency in a murine DOX-induced cardiotoxicity model. These results indicate that *Fgf13* deficiency may protect mitochondria from damage via Parkin. Nevertheless, additional studies are needed to define how FGF13 affects Parkin expression to regulate p53 and apoptosis in DOX-induced cardiotoxicity.

Our study demonstrated that *Fgf13* knockout protects the heart by regulating mitochondrial function via Parkin in a cardiac injury model, although some limitations remain. While mitochondrial damage involves both mitophagy and apoptosis, we focused specifically on the role of FGF13 in Parkin-mediated apoptosis. Although mitochondrial dysfunction manifests through various pathways, such as calcium transport [ 37, 38] and energy metabolism [39], we limited our analysis to changes in the membrane potential. Notably, FGF13 is downregulated in cardiac injury models, but its knockdown confers cardioprotection, which potentially occurs through compensatory mechanisms.

In summary, *Fgf13* deficiency alleviates doxorubicin-induced cardiotoxicity through regulating Parkin-mediated myocardial injury. This study revealed the molecular mechanism underlying the contribution of FGF13 to DOX-induced cardiotoxicity and suggested that FGF13 represents a novel target for the treatment of DOX-induced cardiotoxicity.
